# Intervention to Improve Compliance With National Guidelines on Venous Thromboembolism Chemoprophylaxis for Patients With Operatively Managed Ankle Fractures

**DOI:** 10.31486/toj.22.0038

**Published:** 2022

**Authors:** Lewys Burnett-Jones, Ananth Srinivasan, Alicia Mead, Atul Malik

**Affiliations:** ^1^ Department of Trauma and Orthopaedics, Sandwell and West Birmingham NHS Trust, Birmingham, UK; ^2^ Trauma and Orthopaedics, University Hospitals of Leicester NHS Trust, Leicester, UK; ^3^ Department of Oral and Maxillofacial Surgery, The Dudley Group NHS Foundation Trust, Dudley, UK

**Keywords:** *Ankle fractures*, *anticoagulants*, *enoxaparin*, *orthopedics*, *venous thromboembolism*

## Abstract

**Background:** Trauma and subsequent immobilization of the lower limb increase the risk of venous thromboembolism (VTE). Our aim was to evaluate compliance with national guidance on operatively managed ankle fractures and VTE chemoprophylaxis before and after implementation of a change in practice.

**Methods:** We conducted an initial single-center audit of patients undergoing ankle fracture fixation. The primary outcome was quality of operation note documentation, and the secondary outcome was whether VTE chemoprophylaxis was prescribed on discharge. All stakeholders were educated on audit findings, new guidelines were synthesized, and the practice was re-audited.

**Results:** A total of 137 patients were included in the initial audit, and 49 patients were included in the loop closure. The first audit highlighted that chemoprophylaxis prescription on discharge was significantly higher when both the agent and treatment duration were clearly stipulated in the operation note compared to when either treatment duration or both agent and treatment duration were omitted (97.2% vs 51.8% and 32.4%, respectively, *P*<0.001). Following our intervention, operation note documentation of agent and treatment duration improved from 29% to 90% (*P*<0.001). VTE chemoprophylaxis on discharge significantly improved from 57% to 98% (*P*<0.001).

**Conclusion:** Our closed-loop audit identified suboptimal operation note documentation as the root cause of VTE noncompliance. The operation note is an important clinical interface between the operating theater and ward staff. We addressed these deficiencies with a basic intervention.

## INTRODUCTION

Ankle fractures are common and affect a significant number of people every year. In the United Kingdom, the overall rate of fracture in men aged 50+ years is 71.8/10,000 per year and in women 50+ years, the rate is 155.4/10,000 per year.^1^

The British Orthopaedic Association Standards for Trauma and Orthopaedics (BOASTs) recommend early fixation in the operating theater for patients <60 years of age when the ankle mortise is unstable. Patients >60 years have the option for close contact casts; however, if this treatment cannot achieve reduction, surgery is indicated. The aim of surgery is to achieve reduction and stabilize the ankle mortise.^2^

Trauma and subsequent immobilization of the lower limb increase the risk of venous thromboembolism (VTE).^3^ VTE is a significant burden on morbidity and mortality. A study of 6 major European Union countries estimated the total number of symptomatic VTE events per annum as 465,715 cases of deep vein thrombosis (DVT), 295,982 cases of pulmonary embolism (PE), and 370,012 VTE-related deaths.^4^ A below-knee cast following lower-limb trauma is a significant risk factor for the development of symptomatic VTE within 3 months of application.^5^

A Cochrane systematic review of 8 randomized clinical trials involving 3,680 participants showed that the risk of VTE following lower-limb immobilization was lower in participants who received low molecular weight heparin (LMWH).^6^ Given the evidence, the National Institute for Health and Care Excellence (NICE) recommends consideration of pharmacologic VTE prophylaxis for people undergoing ankle surgery if immobilization is required, total anesthesia time is more than 90 minutes, or the person's risk of VTE outweighs the risk of bleeding.^7^ The BOASTs stipulate that VTE prophylaxis should follow agreed local protocols.^2^

The aims of this study were to investigate the practice of VTE prophylaxis at the Sandwell and West Birmingham (SWBH) National Health Service Trust in accordance with NICE guidelines and BOASTs, compare local practice to national guidance, initiate a quality improvement intervention, and study the results of the intervention.

## METHODS

A 2-cycle single-center quality improvement project was undertaken at SWBH to assess compliance with national guidance. Ethics approval was granted by the SWBH Trust Clinical Effectiveness Team prior to the initial data collection cycle for both cycles of the audit.

The initial data collection cycle was September 2017 to August 2018 and included all patients with operatively managed ankle fractures. We selected this interval because it was the most recent full academic year in relation to project initiation. Data were collected retrospectively. Patients were identified through the SWBH hospital trauma database in which the unique hospital number for all patients undergoing ankle fracture surgery is recorded. We used these hospital numbers to search the hospital electronic patient record system. Data extracted were age, operation note documentation of VTE, discharge prescription of chemoprophylaxis agent, and prescription duration. Exclusion criteria included the following: patients with thromboprophylaxis contraindications (patients already anticoagulated and with high bleeding risk); those with incorrect patient identifiable details in the trauma database; and patients who self-discharged, who died during their inpatient stay, or who had an inpatient stay exceeding 6 weeks. Data were manually entered into an Excel, version 16.53 (Microsoft Corporation) spreadsheet and were manually rechecked after completion. The collected data were analyzed using Microsoft Excel.

The primary outcome measure was whether VTE chemoprophylaxis was documented in the operation note; the secondary outcome measure was whether VTE chemoprophylaxis was prescribed on discharge.

After the first data collection cycle results were analyzed, a Quality Improvement Half-Day (QIHD) was held on February 18, 2019. QIHD is attended monthly by all stakeholders in the Trauma and Orthopaedics department. The stakeholders—the surgeons and the junior doctors on the ward—were educated on the findings of the initial audit, and a local policy based on NICE guidelines^7^ and evidence from the Cochrane review^6^ was agreed upon that stipulated 6 weeks of a once-daily subcutaneous 40 mg injection of enoxaparin for all operatively managed ankle fractures unless contraindicated. The policy was put into writing within 1 week of the QIHD and followed up with an intervention to improve operation note documentation and increase chemoprophylaxis prescribing on discharge. For 3 weeks following the QIHD, posters with the new recommended guidelines were placed in areas of the department that were visible to the ward junior doctors and the operating surgeons completing the operation notes. The requirement for and duration of VTE prophylaxis were further reviewed when the patient attended follow-up clinic with the operating consultant at 2 weeks and 6 weeks.

The authors hypothesized that a new policy stipulating the specific agent and duration of VTE chemoprophylaxis along with education of ward and operating theater staff through departmental teaching at the QIHD and posters in key areas would help improve the consistency of the documentation and discharge VTE chemoprophylaxis prescribing.

The practice was re-audited using the same methodology during a 6-month period from June 2020 to November 2020 at the same hospital. The immediate 6-month period following the QIHD was not selected for re-audit of the practice to ensure that any changes to the practice were long established. The specific period selected was chosen as it was the most recent 6-month period relative to when the decision to re-audit the project was made and to obtain the most up-to-date data set. *P* values were calculated using Fisher exact test.

## RESULTS

The initial data collection cycle (cycle 1) identified 137 operatively managed cases of ankle fracture that met the requirement for VTE chemoprophylaxis. All patients were included in the analysis. The second data collection cycle (cycle 2) identified 50 patients who met the criteria; 1 patient was excluded because of self-discharge.

Operation note documentation of VTE chemoprophylaxis was assessed as follows: (1) full documentation of duration and agent of chemoprophylaxis, (2) nonspecific documentation (some mention of VTE chemoprophylaxis but duration and agent together not specified), and (3) no documentation (agent and duration not specified).

In cycle 1, 29% of cases had both agent and duration specified, 29% had nonspecific documentation, and 42% had no documentation. In cycle 2, operation note documentation of agent and treatment duration improved to 90% (*P*<0.001), 6% had nonspecific documentation, and 4% had no documentation of VTE chemoprophylaxis (Table).

**Table.** Analysis of Primary and Secondary Outcomes by Data Collection Cycle

Outcome	Data Collection Cycle 1, n=137	Data Collection Cycle 2, n=49	*P* Value
Operation note documentation			
Full documentation: agent and duration specified	40 (29)	44 (90)	<0.001
Nonspecific: some mention of VTE chemoprophylaxis but duration and agent together not specified	40 (29)	3 (6)	<0.001
No documentation: no agent or duration specified	57 (42)	2 (4)	<0.001
Discharged with VTE chemoprophylaxis prescription	78 (57)	48 (98)	<0.001

Notes: Data are presented n (%). Data collection cycle 1 was September 2017 to August 2018. Data collection cycle 2 was June 2020 to November 2020. *P* values are calculated using Fisher exact test.

VTE, venous thromboembolism.

The Table also shows the analysis of the secondary outcome. In cycle 1, 57% of cases were discharged with a VTE chemoprophylaxis prescription compared to 98% in cycle 2, a 72% increase (*P*<0.001) in VTE chemoprophylaxis prescribing following the intervention.

During the first cycle, 2 patients had clinically significant VTE events. One patient developed DVT and required lifelong rivaroxaban; the other patient developed a PE, was admitted to the intensive care unit, and required ventilator support. No thrombotic events were identified among the patients in cycle 2.

The Figure demonstrates the relationship between operation note documentation and VTE prescription on discharge during data collection cycle 1. The first audit highlighted that chemoprophylaxis prescription on discharge was significantly higher when both the agent and treatment duration were clearly stipulated in the operation note compared to when either treatment duration or both agent and duration were omitted. The analysis showed that when both the agent and duration were documented in the operation note, 97.2% of patients were discharged with VTE chemoprophylaxis. When the operation note documentation was nonspecific, the discharge VTE chemoprophylaxis rate was 51.8%, and when no documentation was provided, the rate was 32.4%. During data collection cycle 1, chemoprophylaxis prescription on discharge was directly correlated to the agent and treatment duration being documented in the operation note (*P*<0.001).

**Figure.**
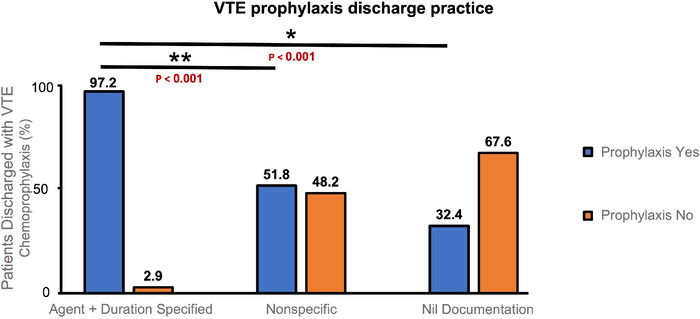
The relationship between operation note documentation and venous thromboembolism (VTE) chemoprophylaxis prescription on discharge during data collection cycle 1. Asterisks denote statistically significant differences.

## DISCUSSION

The initial audit identified that operatively managed ankle fracture operation note documentation was not addressing the issue of chemoprophylaxis for VTE adequately in accordance with the NICE guidelines. In addition, at the time of the initial audit, the department did not have an agreed-upon local protocol for VTE prophylaxis for operatively managed ankle fractures and therefore was not following the BOASTs.

Analysis of the initial data collection cycle showed a strong correlation between operation note documentation and VTE discharge prescriptions. If both agent and duration were documented, discharge VTE chemoprophylaxis was prescribed in most cases, while poor documentation resulted in no VTE chemoprophylaxis being prescribed in the majority of cases. Failure to provide a postoperative chemoprophylaxis prescription for an operatively fixed ankle in 43% of patients during the first cycle could be viewed as poor decision-making. A patient in this cohort developing a postoperative VTE could result in legal consequences because of the lack of intervention.

The identification of the operation note as a key interface between the operating theater and ward staff resulted in a targeted approach to improve documentation. This study shows how a simple intervention can improve the documentation and consequently compliance with national guidance and demonstrates the importance of clear local protocols and staff education on patient safety.

A potential intervention that could further improve the quality of the operation note and compliance with VTE chemoprophylaxis is the use of prepopulated operation notes detailing VTE requirements for the specific operation. However, a limitation of this approach is that the ease of use of prepopulated notes could increase the number of VTE prescriptions given to patients who have absolute contraindications to VTE chemoprophylaxis.

Evidence from the Cochrane systematic review by Zee et al showed that the risk of developing a symptomatic VTE following lower-limb immobilization is 2.1% without the use of LMWH and 0.8% with the use of LMWH, while major adverse events such as hematoma, acute major bleeding, allergic reaction, and thrombocytopenia are rare.^6^ The Cochrane review concluded that moderate quality evidence showed that LMWH was effective in reducing the incidence of VTE in patients with lower-limb cast immobilization compared to no treatment or placebo.^6^ The Cochrane review evidence underpinned the decision by the Trauma and Orthopaedics department to standardize the use of LMWH for all patients following ankle fracture surgery with lower-limb immobilization and to prescribe once daily subcutaneous injections of 40 mg enoxaparin, providing the patient had no absolute contraindication. This simple standardized approach to VTE prophylaxis had the added benefit of being easy to teach and remember for the health care team. In contrast to the conclusions of the Cochrane review by Zee et al, the Prevention of Thrombosis after Lower Leg Plaster Cast (POT-CAST) trial data of 1,435 patients concluded that a prophylactic regime of LMWH during the period of immobilization in patients with lower-limb casting was not effective for the prevention of symptomatic VTE. The authors of the POT-CAST study concluded that more studies are needed to elicit the true effectiveness of LMWH in preventing symptomatic VTEs.^8^

Given that the Cochrane study by Zee et al demonstrated only moderate quality evidence for the use of prophylactic LMWH in preventing VTE^6^ and the POT-CAST trial showed no effect,^8^ an argument can be made for using an increased dose of VTE prophylaxis in selected high-risk patients. One scoring system developed to calculate VTE risk and guide thromboprophylaxis is the TRiP(cast) (Thrombosis Risk Prediction following cast immobilization) score. This approach identifies high-risk patients who would benefit from an increased dose of LMWH and low-risk patients for whom treatment should be withheld to prevent unnecessary adverse incidents.^9^

The results of this study build on existing guidelines that detail both the importance of operation notes and the requirement to stipulate the VTE prophylaxis required, when applicable.^10^

Our study has limitations. We did not account for cases in which the operating team considered VTE chemoprophylaxis, decided it was not indicated, but did not document the decision, so the chemoprophylaxis was not prescribed on discharge. However, medicolegally, no documentation suggests no decision made, and the authors mirrored this approach. Another limitation relates to the scalability of the results given that the study was conducted at a single center.

However, the success of the intervention demonstrates the effectiveness of education in preventing human errors from lack of documentation and prescription.

## CONCLUSION

This study identified the operation note as an important factor in VTE chemoprophylaxis nonprescription. A new local protocol and education of staff significantly improved operation note documentation and discharge VTE chemoprophylaxis. These basic interventions improved adherence to both NICE guidelines and BOASTs. Further improvement may come from the introduction of prepopulated operation notes to ensure 100% compliance with VTE chemoprophylaxis local guidelines.

## Notes

*This article meets the Accreditation Council for Graduate Medical Education and the American Board of Medical Specialties Maintenance of Certification competencies for Patient Care, Medical Knowledge, and Practice-Based Learning and Improvement.*
